# Risk factors for tick attachment in companion animals in Great Britain: a spatiotemporal analysis covering 2014–2021

**DOI:** 10.1186/s13071-023-06094-4

**Published:** 2024-01-22

**Authors:** Elena Arsevska, Tomislav Hengl, David A. Singleton, Peter-John M. Noble, Cyril Caminade, Obiora A. Eneanya, Philip H. Jones, Jolyon M. Medlock, Kayleigh M. Hansford, Carmelo Bonannella, Alan D. Radford

**Affiliations:** 1 Unit for Animals, Health, Territories, Risks and Ecosystems (UMR ASTRE), French Agricultural Research Centre for International Development (CIRAD), 34980 Montferrier-sur-Lez, France; 2 OpenGeoHub Foundation, 6708 PW Wageningen, The Netherlands; 3 Institute of Infection, Veterinary and Ecological Sciences, University of Liverpool, CH64 7TE Neston, UK; 4 Earth System Physics Department, Abdus Salam International Centre for Theoretical Physics (ICTP), 34151 Trieste, Italy; 5 Health Programs, The Carter Center, 30307 Atlanta, Georgia, USA; 6 Medical Entomology and Zoonoses Ecology, UK Health Security Agency, SP4 0JG Salisbury, UK; 7 NIHR Health Protection Research Unit in Environmental Change and Health, WC1E 7HT London, UK; 8 Laboratory of Geo-information Science and Remote Sensing, Wageningen University & Research, 6708 PB Wageningen, The Netherlands

**Keywords:** Ticks, Risk factors, Ensemble machine learning, Space-time, Companion animals, Electronic health records, Great Britain

## Abstract

**Background:**

Ticks are an important driver of veterinary health care, causing irritation and sometimes infection to their hosts. We explored epidemiological and geo-referenced data from > 7 million electronic health records (EHRs) from cats and dogs collected by the Small Animal Veterinary Surveillance Network (SAVSNET) in Great Britain (GB) between 2014 and 2021 to assess the factors affecting tick attachment in an individual and at a spatiotemporal level.

**Methods:**

EHRs in which ticks were mentioned were identified by text mining; domain experts confirmed those with ticks on the animal. Tick presence/absence records were overlaid with a spatiotemporal series of climate, environment, anthropogenic and host distribution factors to produce a spatiotemporal regression matrix. An ensemble machine learning spatiotemporal model was used to fine-tune hyperparameters for Random Forest, Gradient-boosted Trees and Generalized Linear Model regression algorithms, which were then used to produce a final ensemble meta-learner to predict the probability of tick attachment across GB at a monthly interval and averaged long-term through 2014–2021 at a spatial resolution of 1 km. Individual host factors associated with tick attachment were also assessed by conditional logistic regression on a matched case–control dataset.

**Results:**

In total, 11,741 consultations were identified in which a tick was recorded. The frequency of tick records was low (0.16% EHRs), suggesting an underestimation of risk. That said, increased odds for tick attachment in cats and dogs were associated with younger adult ages, longer coat length, crossbreeds and unclassified breeds. In cats, males and entire animals had significantly increased odds of recorded tick attachment. The key variables controlling the spatiotemporal risk for tick attachment were climatic (precipitation and temperature) and vegetation type (Enhanced Vegetation Index). Suitable areas for tick attachment were predicted across GB, especially in forests and grassland areas, mainly during summer, particularly in June.

**Conclusions:**

Our results can inform targeted health messages to owners and veterinary practitioners, identifying those animals, seasons and areas of higher risk for tick attachment and allowing for more tailored prophylaxis to reduce tick burden, inappropriate parasiticide treatment and potentially TBDs in companion animals and humans. Sentinel networks like SAVSNET represent a novel complementary data source to improve our understanding of tick attachment risk for companion animals and as a proxy of risk to humans.

**Graphical Abstract:**

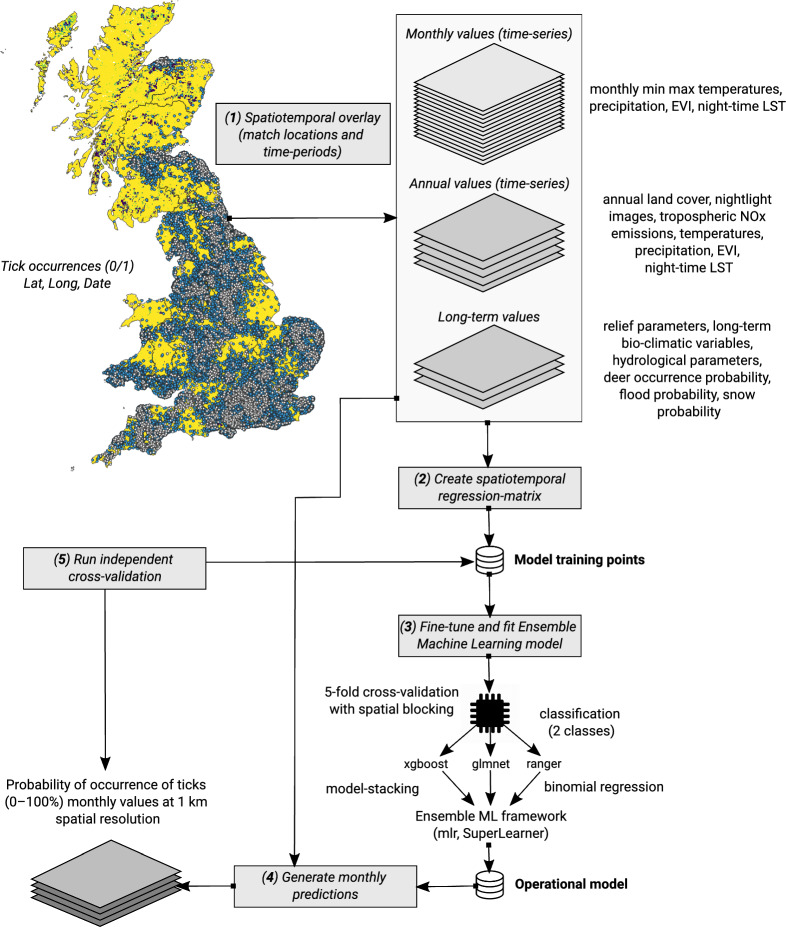

**Supplementary Information:**

The online version contains supplementary material available at 10.1186/s13071-023-06094-4.

## Background

According to recent estimates, approximately 11.6 million dogs and 10.1 million cats are kept as companion animals in Great Britain (GB) [1]. These companion animals are frequently infested with ticks [2, 3], some of which (*Ixodes spp*.) can be important vectors of tick-borne diseases (TBD) such as *Borrelia burgdorferi* sensu lato (s.l.) causing Lyme borreliosis in cats, dogs and humans and tick-borne encephalitis (TBE) virus causing TBE in dogs and humans [4]. Consequently, there is a *“One health”* imperative to understand the risk factors contributing to tick attachment in companion animals, not just for animal welfare, but also as a proxy of risk to humans [5, 6].

The likelihood of tick attachment for any host species is largely driven by local tick presence and abundance, which in turn is dependent on several factors such as habitat type, climate and host availability [7–9]. However, host behaviour is also important. As such, there may be established populations of ticks lacking exposure to particular hosts or, conversely, low numbers of ticks with high exposure to companion animals, the latter being more important for the risk assessment of tick attachment [10, 11].

Two large-scale GB surveys have assessed tick risk in companion animals. The first, mainly cross-sectional in nature, assessed tick distribution and host risk factors associated with tick attachment based on subsets of animals identified by practitioners as having ticks following a specific enhanced clinical examination regime; molecular methods were also used to detect TBD pathogens in ticks removed from sampled cats [2, 3, 12]. The second survey, the Tick Surveillance Scheme (TSS) maintained by the United Kingdom (UK) Health Security Agency, records the distribution, seasonality and host associations of ticks submitted for identification by members of the public, veterinarians and other professionals across the UK [10, 13]; between 2010 and 2016, samples from dogs and cats made up 39.1% and 14.1% of all submitted ticks, respectively [13].

In addition to these approaches, we have explored electronic health records (EHRs) as a novel and perhaps complementary method to assess companion animal health [14]. EHR analysis has the advantage of being able to assess the health status of animals in near real-time and over time while minimising the costs of epidemiological and entomological fieldwork. Using such an approach, Tulloch et al., [15] provided a first mapping of the spatiotemporal (postcode areas and season) tick “activity” in companion animals using over 2000 EHRs in which veterinary health professionals participating in the Small Animal Veterinary Surveillance Network (SAVSNET) recorded tick attachment to, or removal from, their patients. Since 2014, SAVSNET has used this large network of veterinary clinics across GB to continue to describe spatiotemporal trends in tick activity in companion animals (https://www.liverpool.ac.uk/savsnet/real-time-data/).

Despite these studies, there remains a need to understand better how exposure, combined with environmental, climate, anthropogenic and host distribution factors, can inform models capable of predicting areas at higher risk for tick attachment in companion animals and as a proxy for a risk to humans [6]. The need for such predictive models stems from an inherent inability of any surveillance system to sample all areas evenly. Therefore, in this study, we build on our earlier work [15] and explore whether an expanded dataset of EHRs from SAVSNET collected between 2014 and 2021, representing one of the largest datasets of ticks known from EHRs, combined with epidemiological data on the host and environmental, climate, anthropogenic and host distribution factors, can be used to identify the main individual and spatiotemporal drivers for tick attachment and predict risk for tick attachment in GB. We used an ensemble machine learning framework to provide temporal and spatial estimates of the probability of tick attachment that can serve as proxies for areas and months for tick attachment in companion animals and as a proxy of risk to humans across GB.

## Methods

### Electronic health records

SAVSNET EHRs were collected between the 1 April 2014 and 31 December 2021 from 452 veterinary clinics, representing approximately 18% (452/ 2483) of the registered small animal veterinary clinics across GB. Each EHR contains a broad range of information relating to the animal (sex, species, breed, date of birth, neuter status), consultation date, pet owner postcode and a free text clinical narrative and generally relates to a single veterinary visit by a single animal. This information is supplemented by a practitioner-derived main presenting complaint for each consultation, chosen from ten broad categories (gastroenteric, renal, pruritus, respiratory, trauma, tumour, health check-up, vaccination and other non-specific consultations).

For assessing the individual risk factors for tick attachment, age at consultation was divided into six classes: age < 1 year old (kitten/puppy), 1 to 2 (junior), 2 to 6 (adult), 6 to 10 (mature), 10 to 14 (senior) and > 14 years at consultation (geriatric). Breeds were categorised into either registered breed groups: in cats, Asian, Mediterranean or Western European breed group [16, 17]; in dogs, gundog, hound, pastoral, terrier, toy, utility or working breed group [18, 19]; and crossbreeds and breeds not recorded/recognisable (unclassified) for both cats and dogs. Breeds were also classified based on coat length as short, long, semi-long (medium) or not recorded/unknown (unclassified) for cats [20, 21] and dogs [18, 19].

Each EHR was geo-referenced (latitude/longitude) using the pet owner’s postcode linked to the GB National Statistics Postcode Directory. This spatial location of the pet owner’s address may not necessarily match the location where the dog or cat acquired the tick. Therefore, for spatiotemporal modelling, we worked at a 1-km spatial resolution and considered that the actual location of tick attachment, i.e. the outdoor location of exposure to ticks, matched the 1-km grid cell of the pet owners’ home [22], as most dog owners walk their pets most frequently near their homes [23]. Similarly, most domestic cats’ roaming ranges are close to, and inevitably centred on, their home [24]. We further used the 1-km grid cell of the pet owners’ home as a proxy for an urban, suburban or rural cat or dog [25]. More precisely, urban included dense urban areas with little vegetation, such as town and city centres. Suburban included suburban areas with a mix of urban and vegetation signatures. The remaining were considered rural areas [25].

The 1-km grid was obtained from the Ordnance Survey National Grid (Digimap Service, University of Edinburgh at http://digimap.edina.ac.uk, 1 January 2015). All spatial data described in the manuscript were (re-)projected to fit the British National Grid datum, EPSG 27700. Where relevant, the regionalization of GB was defined according to the Nomenclature of Territorial Units for Statistics (NUTS) regions.

Data collection and use by SAVSNET were ethically approved by the University of Liverpool Research Ethics Committee (RETH000964).

### Tick records

A tick presence record (further referred to as 'tick record') was defined as an EHR in which a veterinary surgeon or nurse recorded tick attachment and/or removal during a consultation. Narratives recording prior removal of ticks by owners were not included in the analyses, as owners often incorrectly identified pathologies (warts, cysts) and anatomy (nipples) as ticks (authors unpublished observations). In brief, a simple free text filter was used to identify clinical narratives containing the words *‘tick’* or *‘ticks’*, whilst excluding the word *’stick’*; domain experts read the resulting narratives to confirm the observation of a tick by the health professional was described in the narrative [15]. The remaining EHRs not found by the free text filter were considered tick absence records; we emphasise that such records did not necessarily mean a tick was truly absent from a given animal since this could not be confirmed from the free text narrative of the EHR.

### Spatial covariates

For spatiotemporal modelling we used 75 environmental (habitat and vegetation), climate, anthropogenic and host distribution covariates (Additional file 5: Table S1), chosen according to the biology and ecology of *Ixodes ricinus* and *I. hexagonus* [7–9], the two major tick species recorded in cats and dogs in GB [2, 3], which we briefly outline below.

The Castor bean (deer/sheep) tick, *I. ricinus*, is an exophilic tick. It actively quests for hosts; consequently, its presence is strongly associated with specific environmental and climatic conditions. Common habitats tend to be lowland, humid areas such as unmanaged grasslands, forest edges and woodlands with sufficiently dense undergrowth, though they can also be found in (peri-)urban and recreational parks [26, 27]. Immature *I. ricinus* feed on small and larger mammals, birds and lizards, whereas adults feed on large ruminants (sheep, cattle and deer) but also cats, dogs and wild carnivores [13, 28]. It has been recorded throughout GB. It usually displays a bimodal pattern of activity, with a peak between April and July [10, 13].

The Hedgehog tick, *I. hexagonus*, is an endophilic tick. It spends all life cycle stages inside burrows or nests of their hosts; free-ranging ticks are rarely encountered. It is a specialist parasite, with hedgehogs the dominant host (*Erinaceus* spp.), followed by small carnivorous mammals (dogs, cats, foxes) and other animals with a permanent dwelling [8, 13, 28]. Hedgehogs live in a broad range of habitats, particularly woodland edges, hedgerows and suburban habitats, where they can encounter companion animals [9, 13]. *Ixodes hexagonus* has been recorded throughout GB [10, 13], though most observations are from England. Being a nidicolous tick, *I. hexagonus* shows fewer seasonal changes than *I. ricinus*; whilst records are highest in spring and summer, they are recorded all year [9, 13].

#### Habitat and vegetation

Ticks are commonly associated with broadleaf or mixed woodland, although high densities can be found in (peri-)urban parks and recreational sites [29, 30]. Inhabited by different mammalian species (e.g. deer, rodents, hedgehogs), these habitats create optimal conditions sustaining the tick life cycle [7–9]. Therefore, we used the annual land cover fractions for the classes of broadleaf woodland, coniferous woodland, natural grassland and inland marshes [31], the annual percentage of cropland [32] and long-term flood occurrence risk [33] to see how different land covers determine the likelihood of ticks coming into contact with companion animals/humans and thus attachment. Ticks are also sensitive to local environmental conditions, such as the thickness of the tree canopy or soil moisture at the ground level [7]. We used four descriptions of the Enhanced Vegetation Index (EVI: monthly mean, annual minimum, mean and maximum EVI) to explore the global range of vegetation greenness and density. EVI values range from -1 to +1, with higher numbers indicating a higher density of green vegetation [34]. We also used the Topographic Wetness Index to characterize rainfall runoff patterns, areas of potential increased soil moisture and ponding areas [35]. Gilbert et al., 2010 [36], found a negative association between tick densities and higher altitudes; thus, we used elevation and associated topographic indicators, such as slope, northness and eastness [35], to assess finer-scale conditions related to topography [37].

#### Climate

Temperature influences the beginning of the tick questing season, development rate of the tick population and chances of survival through winter [7]. Milder winters/warm springs can result in early questing ticks. Hot summers can result in increased development from one life stage to the next [9]. Ticks also require a relative humidity of at least 80% with moderate to high rainfall with good vegetation (i.e. litter layer and soil that remain humid during the day). Rain is necessary during the summer, but drought and heavy rain may prevent the development of new tick populations [7]. We therefore used the monthly minimum and maximum temperature based on the TerraClim dataset [38] and the long-term minimum, mean and maximal monthly quantiles of nighttime land surface temperature based on the MODIS dataset [39]. We also used monthly precipitation data and long-term Bioclimatic variables (bioclim 1-7, bioclim 10-12) based on the CHELSA climate time series dataset [40, 41].

*Ixodes ricinus* overwinters on the ground and snow cover can enhance survival, which may prevent ground temperatures from falling below zero [42]. We, therefore, also included the monthly maximal and standard deviation of snow probability for GB [43].

To account for the seasonality of weather throughout the year (i.e. the interchange of cold and warm months), we also calculated the cosine of the month of the year and used it further as a spatial covariate.

#### Human-induced factors

Urbanisation creates specific environmental conditions (less green space, warmer temperature, dust and air pollution, night lights) that alter tick abundance and activity [44]. We, therefore, used the annual Human Footprint Index (HFP) [45], the monthly Sentinel-5P Tropospheric (minimum, mean, maximum values) Nitrogen Dioxide Density—a traffic-related air pollutant [46]—and the annual nighttime lights (minimum, mean, maximum values) [47] as measures of the direct and indirect human pressure on the environment, related to urbanisation, that can potentially drive specific patterns for tick attachment in a given area.

#### Host distribution

We also included predictor variables for cat and dog distributions in GB [1]. Since deer are important hosts and reservoirs, driving tick abundance and facilitating their dispersal [7], we also included a covariate summarising the mean probability of the presence of red, roe and fallow deer in each 1-km grid cell [48, 49].

### Individual risk factors for tick attachment

To assess the effects of sex, neuter status, age, breed and coat length on the odds of tick attachment, a separate matched case-control study for both cats and dogs was undertaken. For each tick record (case), we selected three records where tick attachment was not recorded (control) during a health visit in the same week and veterinary clinic using the MatchIt package (version 4.5.4) [50] in the R programming language (version 4.3.1; further referred to as R) [51].

Conditional logistic regression was performed on the matched dataset, using the R package survival (version 3.5.5) [52], accounting for the matching criteria (week of visit and veterinary clinic) as the model’s strata. Tick attachment status (presence/absence) at consultation was considered a binary outcome variable. An initial multivariable conditional logistic regression model included variables associated ($\hbox {p}<0.2$) with tick attachment status on univariable conditional logistic regression analysis. Variables significantly ($\hbox {p}<0.05$) associated with tick attachment status were retained in a final multivariable conditional logistic regression model. A backward selection process was utilized to produce a model fit with the lowest Akaike information criterion (AIC). Adjusted odds ratios (OR) and their 95% confidence intervals (CI) were calculated from the final conditional logistic regression model parameter estimates by accounting for the model’s strata.

### Spatiotemporal risk factors for tick attachment

#### Exploratory spatial analysis

We first created kernel ratio maps of the two species records (i.e. cats and dogs) compared to all records (as point data) to estimate whether the two species displayed the same spatial distribution. Next, we compared the distributions with the spatial locations of the SAVSNET veterinary clinics to check for spatial biases in the point data.

Second, to identify areas that contain a higher density of tick records than would be expected with complete spatial randomness whilst accounting for the underlying distribution of the point data, we estimated the spatial relative risk (RR) given the relative densities of the tick presence and absence records (as point data). An adaptive bandwidth was used to compensate for potential over-smoothing in dense areas, calculated symmetrically for tick presence and absence records. Asymptotic tolerance contours of *p*-values ($\hbox {p}<0.05$) were mapped to show statistically significant areas of elevated risk for a higher density of tick records [53]. The kernel ratio maps and RR were estimated using the R package sparr (version 2.3.10) [54]. Visually, the preliminary exploratory analysis of the point observations of the tick records in cats and dogs showed no difference; thus, we did the spatiotemporal modelling on cats and dogs together.

#### Spatiotemporal modelling

**Fig. 1:**
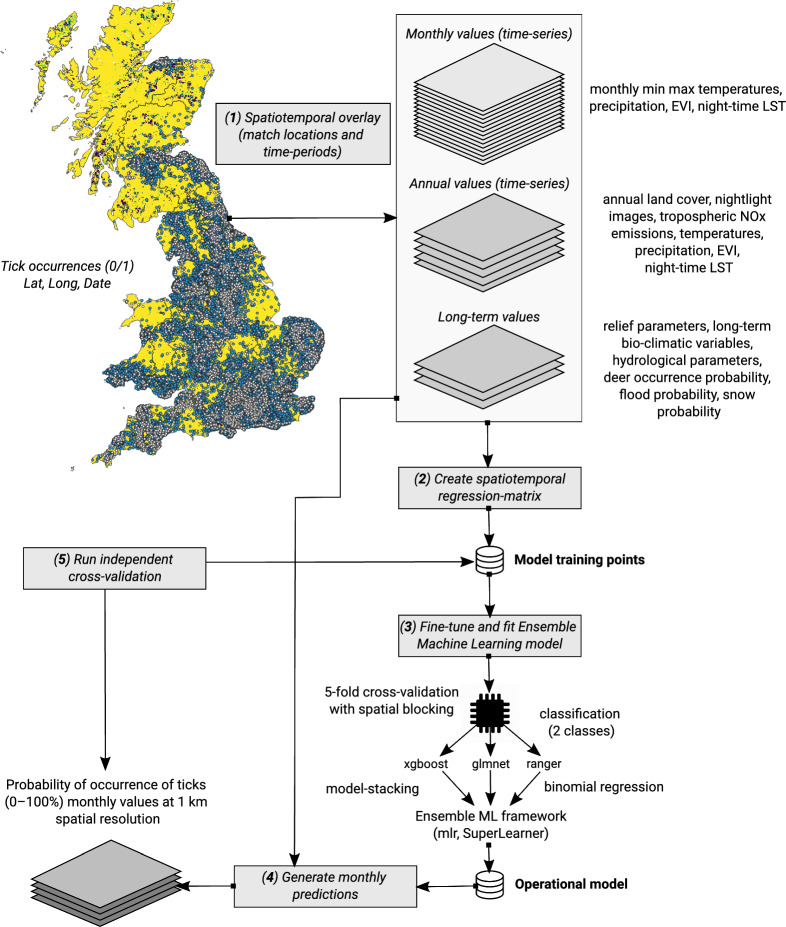
General computational scheme used to produce spatiotemporal predictions (monthly probabilities for tick attachment) at 1-km spatial resolution. The map shows locations of tick presence records (point observations in white) versus tick absence records (point observations in blue) in companion animals from the SAVSNET network in Great Britain between 2014 and 2021

We have built an ensemble machine learning (ML) model inspired by the work of Bonannella et al., [55]. The modelling workflow consisted of five main steps (Fig. 1): (i) overlay 1-km grid cells with the time series of covariate layers using their spatial (longitude and latitude of the centroid) and temporal (date of consultation) reference of the record (tick presence/absence), (ii) create a spatiotemporal classification/regression matrix with all observations (tick presence/absence) and covariate values; (iii) fit an ensemble ML model to predict the probability of tick attachment; (iv) generate the predictions and (v) visualise and run validation of accuracy. Output predictions were time series of images showing monthly changes in the probability of tick attachment between 2014 and 2021 at a 1-km grid. Monthly predictions have been further averaged to show the long-term risk of tick attachment through 2014–2021.

The ensemble ML model was based on stacking three independently fitted ML algorithms (learners) [56], commonly used for spatial modelling:

- class.ranger: fully scalable implementation of Random Forest (RF) [57] and often preferred algorithm of choice because it gives good results with little fine-tuning and adaptation, particularly for non-normal and multicollinear data [58, 59]. RF algorithm was previously used for spatial mapping of the risk of ticks in livestock farms in GB [58] and mapping of tick dynamics using volunteer data of collected ticks in The Netherlands [59].
- class.xgboost: regularized implementation of Gradient-boosted Trees (GBT) [60, 61] with similar characteristics to RF. Boosted regression tree models were used for mapping the risk of TBE in mainland China [62] and prediction of the human risk of exposure to *I. ricinus* in several Scandinavian countries [63].
- class.glmnet: logistic regression with Generalized Linear Model (GLM-net) with Lasso regularization [64], widely used for ecological niche modelling and spatial disease mapping such as ticks in Norway and TBE in Europe [37, 65].

We used ensemble ML since it can be considered a remedy for potential model over-fitting. In the case of spatially clustered samples (in this study, records can be considered biased to urban/suburban areas), spatial blocking, i.e. spatial cross-validation during model training, helps reduce potential over-fitting that can be significant [66]. Note that the records in our study followed a zero-inflated distribution, with the vast majority of observations being 0 values ($>99.84\%$), indicating “tick absence” at the point location (consequently at a 1-km grid level), and autumn and winter months are often without tick records. Such a skewed distribution did not prevent us from fitting models, especially RF and GBT algorithms that can handle extreme disbalances between 0 and 1 values [59]. For predicting the risk for tick attachment, we have set the density of dogs and cats to the 5th percentile so that the final produced maps do not contain the potential spatial bias mentioned above.

We run the ensemble ML model fitting and prediction in four steps, as implemented in the R package mlr (version 2.19.1) [67]: (i) hyper-parameter fine-tuning: we first determined the number of covariates (predictors) to split on each node (i.e. mtry) for class.ranger and class.xgboost parameters by iterative fine-tuning; (ii) Training: we trained the three learners independently using five-fold cross-validation with spatial blocking at $5\times 5~\hbox {km}$; (iii) stacking: the out-of-fold predictions of the three learners were then used as a training set to fit a second-level model (i.e. *meta-learner*); (iv) after model fitting, we produced predictions by using the second-level model (binomial regression) based on three base learners.

To assess the contribution of each predictor variable in maximizing model performances, we calculated and plotted the variable importance of the best learner of the ensemble model. The method used to calculate the variable importance for the RF was Gini importance; for GBT, we used the gain metric and, for GLM, the coefficients for the minimum fitted value of $\lambda$. For most tree-based methods, such as RF and GBT, multicollinearity is not an issue, i.e. it does not impact the final model performance or lead to bias [59]; thus, we used it for modelling all initial covariates.

We derived a partial dependence plot (PDP) for the most important covariates obtained with the variable assessment to interpret the prediction from the ensemble ML model outputs. More precisely, the PDP shows the marginal effect of each covariate on the predicted outcome of the ML model. PDP shows whether the relationship between the target and a covariate is linear, monotonic or more complex. A flat PDP indicates that the covariate is unimportant, and the more the PDP varies, the more important the covariate is. The sum of the variable importance for all covariates equals 1. The partial dependence plots were derived using the R package pdp (version 0.8.1) [68].

Final predictions were delivered as probability maps of tick attachment risk and model uncertainty maps. We considered as model uncertainty the standard deviation of the predicted values of the base learners.

The predictive performance of the spatiotemporal ensemble ML model was assessed through five-fold spatial cross-validation repeated five times for a total of 25 repetitions, using the area under the receiver-operating characteristic (ROC) curve (AUC) as a performance metric. Historically, methods favouring threshold selection have been more widely used for assessing binary classification problems, particularly spatial distribution modelling [69]. In this case, a threshold was arbitrarily selected, with 0.5 being the threshold selected by default. The predicted values were assigned to one of the two classes based on the predicted probability value. However, the AUC is a threshold-independent performance metric; constructing the ROC curves to calculate the AUC uses all possible thresholds to classify the predicted values into confusion matrices. The sensitivity of each matrix is then compared against the proportion of false positives, thus avoiding using just one of the thresholds to evaluate model performance. AUC values close to 1 indicate high model performance, while AUC values close to or below 0.5 indicate poor or worse performance than a random classifier.

## Results

### Descriptive analysis of the tick records

In total, 2,109,012 cat and 5,409,697 dog EHRs were collected from 2,173,364 unique animals during the study period (1 April 2014–31 December 2021). There were 3295 tick records in cats and 8446 in dogs, representing 0.16% of SAVSNET EHRs. Overall, tick records (1 to 9 records) were observed in 6364 of the 238,747 1-km grid cells that make up GB (2.67%). Most tick records were in suburban (52.66% in cats and 53.24% in dogs) and rural areas (44.01% in cats and 41.64% in dogs). Only 3.34% of tick records in cats and 5.11% in dogs were in urban areas (Table 1).

**Table 1:** Epidemiological overview of the tick records of the SAVSNET cat and dog population in Great Britain from 2014 to 2021

Variable	Category	Number (%) of cats	Number (%) of dogs
Reason for consultation	Gastroenteric	17 (0.52)	94 (1.11)
	Kidney disease	8 (0.24)	8 (0.09)
	Health-check-up	1406 (42.67)	3887 (46.02)
	Non-specific unwell	604 (18.33)	1761 (20.85)
	Post-operative check-up	98 (2.97)	306 (3.62)
	Pruritus	65 (1.97)	265 (3.39)
	Respiratory	12 (0.36)	12 (0.14)
	Trauma	214 (6.49)	292 (3.46)
	Tumour	18 (0.55)	124 (1.47)
	Unknown reason for consult	3 (0.09)	14 (0.17)
	Vaccination	850 (25.8)	1662 (19.68)
Age	< 1 year (kitten/puppy)	224 (6.80)	1402 (16.60)
	1–2 years (junior)	386 (11.71)	1096 (12.98)
	2–6 years (adult)	1046 (31.75)	2872 (34.0)
	6–10 years (mature)	811 (24.61)	2056 (24.34)
	10–14 years (senior)	572 (17.36)	911 (10.79)
	> 14 years (geriatric)	256 (7.77)	109 (1.29)
Sex	Female	1230 (37.33)	3850 (45.58)
	Male	2065 (62.67)	4596 (54.42)
Neuter status	Entire	526 (15.96)	2635 (31.20)
	Neutered	2769 (84.04)	5811 (68.80)
Recognised breed group	Recognised breed	285 (8.65)	5773 (68.36)
	Crossbreed	2772 (84.13)	2535 (30.01)
	Unclassified	238 (7.22)	138 (1.63)
Coat length	Short	2334 (70.83)	2093 (24.78)
	Semi-long	114 (3.46)	3211 (38.02)
	Long	517 (15.69)	900 (10.66)
	Unclassified	330 (10.02)	2242 (26.54)
Season of occurrence	Spring	1279 (38.82)	2374 (28.11)
	Summer	1001 (30.38)	4610 (54.58)
	Autumn	736 (22.34)	1256 (14.87)
	Winter	279 (8.47)	206 (2.44)
Owner residence location	Urban	110 (3.34)	432 (5.11)
	Suburban	1735 (52.66)	4497 (53.24)
	Rural	1450 (44.01)	3517 (41.64)
NUTS level 1	North East (England)	174 (5.28)	815 (9.65)
	North West (England)	171 (5.19)	481 (5.70)
	Yorkshire and The Humber	208 (6.31)	695 (8.23)
	East Midlands (England)	112 (3.40)	325 (3.85)
	West Midlands (England)	129 (3.92)	322 (3.81)
	East of England	336 (10.20)	813 (9.63)
	Greater London	31 (0.94)	90 (1.07)
	South East (England)	1238 (37.57)	2638 (31.23)
	South West (England)	679 (20.61)	1542 (18.26)
	Wales	72 (2.19)	193 (2.29)
	Scotland	145 (4.40)	532 (6.30)
Total		3295	8446

**Fig. 2:**
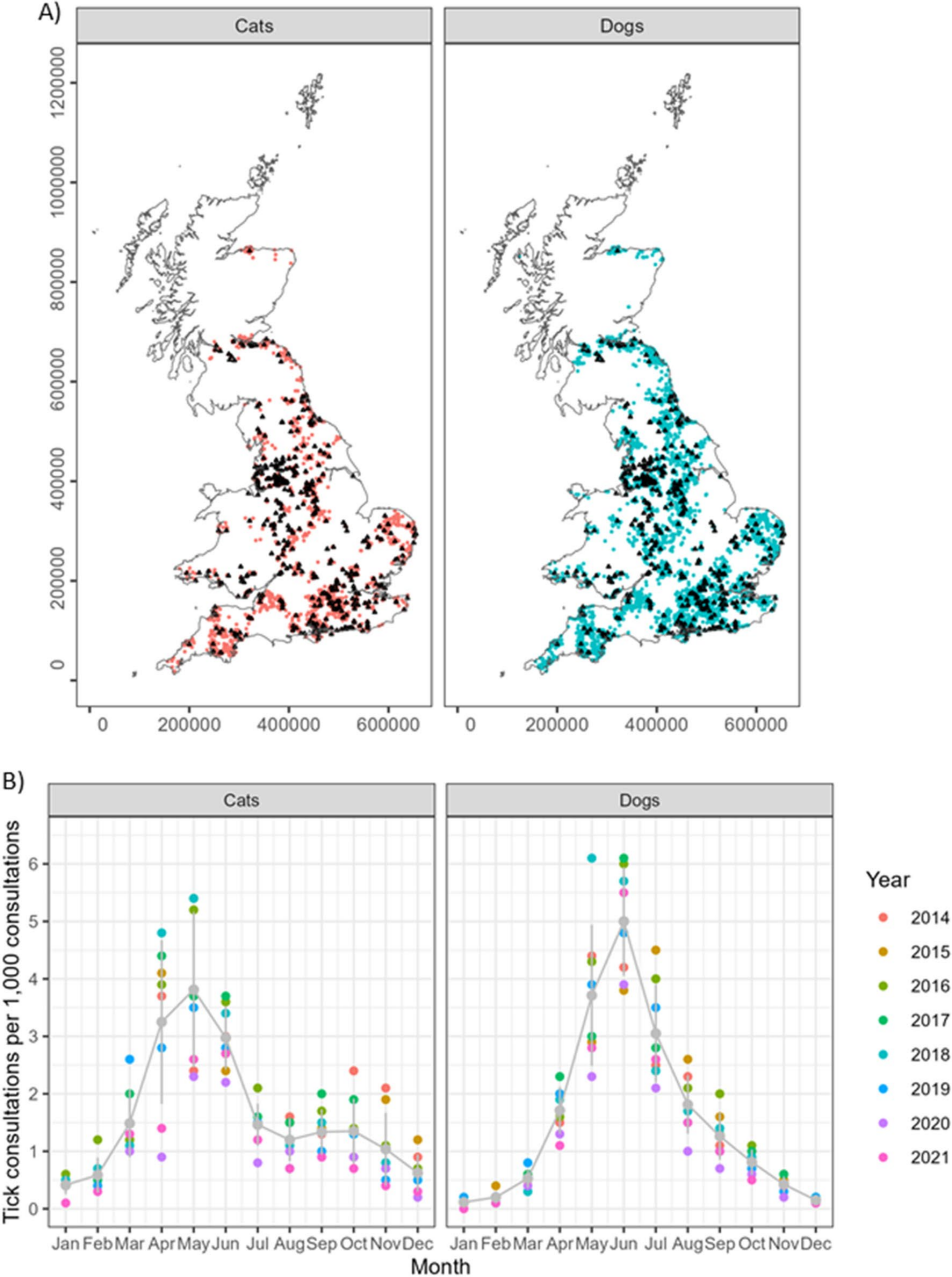
**A** Distribution of tick presence records among SAVSNET cats (red) and dogs (cyan) in Great Britain between April 2014 and December 2021 and their contributing veterinary clinics (black). **B** Seasonality of tick presence records by month and year, plotted as the number of tick records per 1000 SAVSNET cat and dog consultations, respectively, in a given month and year. The grey point line shows the mean across all years, including the standard deviation around this mean

Tick records were observed across GB during all months and seasons (Table 1). Overall, the highest numbers were in areas in South East England (37.57% in cats, 31.3% in dogs), South West England (20.61% in cats, 18.26% in dogs) and the coast of East of England (10.20% in cats, 9.63% in dogs), followed by the southern part of Yorkshire and Humber (6.31% in cats, 8.23% in dogs) and coastal areas in North East England (5.28% in cats, 9.65% in dogs) (Fig. 2A, Table 1).

There was no visual difference in the distribution ratio of cat and dog records (Additional file 2: Figure S2). The RR and tolerance contours ($\hbox {p}<0.05$) showed that veterinary clinics in areas in South East and South West England, East of England, North West, and South and North Eastern Scotland recorded significantly more ticks in both cats and dogs (Additional file 3: Figure S3).

In cats, ticks were mainly observed in spring (38.82%), peaking in May (17.03%, 4 tick records per 1000 consultations), with a second lower peak in September (8.41%, 1.4 per 1000 consultations) (Table 1, Fig. 2B). In dogs, the peak was in summer (54.58%), particularly in June (26.86%, 5.3 per 1000 consultations). The fewest ticks were recorded in January (cats 72; 0.4% per 1000 consultations and dogs 52; 0.1% per 1000 consultations, respectively).

Ticks were mainly observed during routine health checks (42.67% in cats and 46.02% in dogs). Most affected animals were adults, between 2 and 6 years old (31.75% EHRs in cats and 34.00% dogs). In both species, males were more frequently affected (62.67% in cats and 54.42% in dogs). Most animals affected were neutered (84.04% of cats and 68.80% of dogs) (Table 1). Regarding breeds, 84.13% of tick records in cats were crossbreeds, while 68.36% of tick records in dogs were in recognised breeds. Ticks were mainly recorded in short-hair cats (70.83%) and medium (semi-long) haired dogs (38.02%).

### Individual host risk factors for tick attachment

**Fig. 3:**
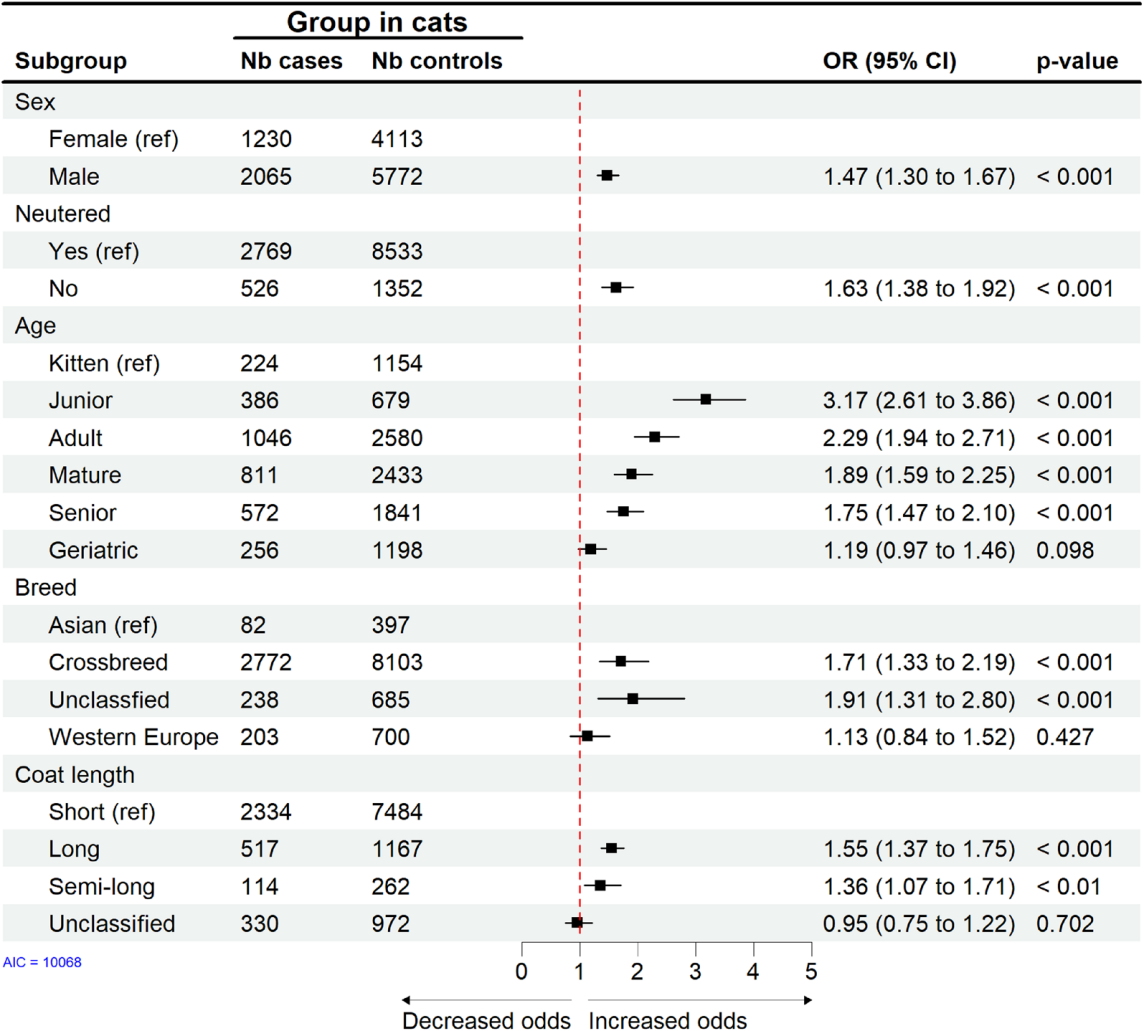
Cats. Final multivariable conditional logistic regression model for risk factors associated with tick attachment in cats based on 13,180 EHRs. Nb = number, Ref = reference, OR = odds ratio adjusted to week of visit and veterinary practice, CI = confidence interval

**Fig. 4:**
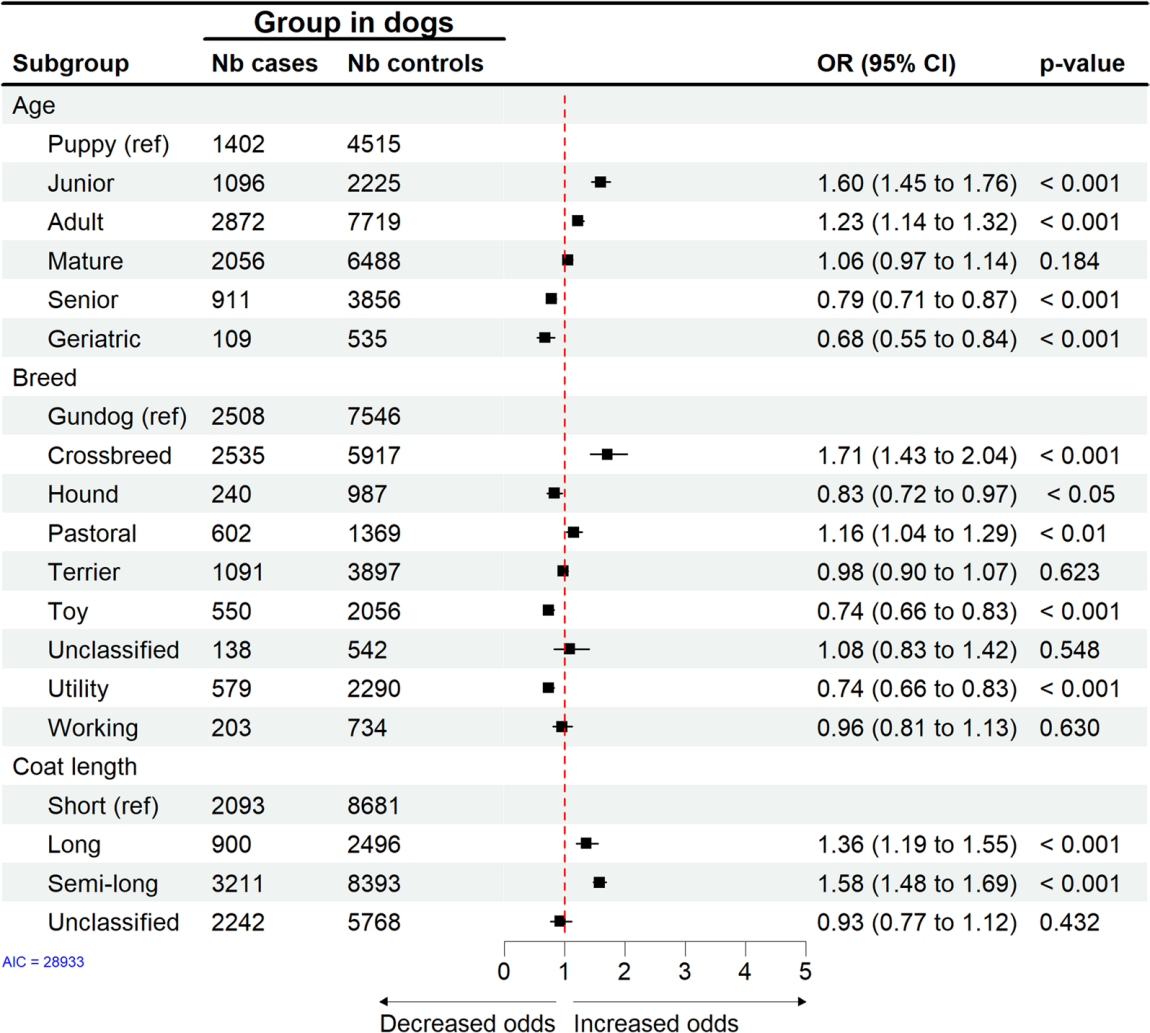
Dogs. Final multivariable conditional logistic regression model for risk factors associated with tick attachment in dogs based on 33,784 EHRs. Nb = number, Ref = reference, OR = odds ratio adjusted to week of visit and veterinary practice, CI = confidence interval

Compared to kittens and puppies, tick attachment in both cats (Fig. 3) and dogs (Fig. 4) was highest among animals of 1 to 2 years of age, i.e. juniors ($\hbox {OR}=3.17$; 95% CI: 2.61$-$3.86; $\hbox {p}<0.001$ in cats, $\hbox {OR}=1.60$; 95% CI: 1.45$-$1.76; $\hbox {p}<0.001$ in dogs) and adult cats and dogs between 2 and 6 years of age ($\hbox {OR}=2.29$; 95% CI: 1.94$-$2.71; $\hbox {p}<0.001$ in cats, $\hbox {OR}=1.23$; 95% CI: 1.14$-$1.32; $\hbox {p}<0.001$ in dogs). The risk of recorded tick attachment decreased with age in both species. Between species, the odds for recorded tick attachment in young cats were twice as high compared to dogs.

In cats, males and not neutered animals ($\hbox {OR}=1.47$; 95% CI: 1.30$-$1.67; $\hbox {p}<0.001$ and $\hbox {OR}=1.63$; 95% CI: 1.38$-$1.92; $\hbox {p}<0.001$, respectively) had higher odds for tick attachment. In contrast, in dogs, neither sex nor neuter status was a significant predictor for tick attachment.

In cats and dogs, unclassified/unknown breed groups and crossbreeds had the highest odds for tick attachment ($\hbox {OR}=1.91$; 95% CI: 1.31$-$2.80; $\hbox {p}<0.001$ in cats and $\hbox {OR}=1.71$; 95% CI: 1.43$-$2.04; $\hbox {p}<0.001$ in dogs). In dogs, compared to the gundog breed group, the pastoral breed group had an increased risk of tick attachment ($\hbox {OR}=1.16$; 95% CI: 1.04$-$1.29; $\hbox {p}<0.01$), while toy and utility breed groups had decreased odds for tick attachment.

In cats and dogs, the highest odds for tick attachment were in animals with long and medium/semi-long coat length ($\hbox {OR}=1.55$; 95% CI: 1.37$-$1.75; $\hbox {p}<0.001$ in cats, $\hbox {OR}=1.56$; 95% CI: 1.48$-$1.69; $\hbox {p}<0.001$ in dogs).

### Spatiotemporal risk factors for tick attachment

The ensemble ML model had moderate to high performance; according to the repeated five-fold spatial cross-validation results, the AUC was 0.80 (Additional file 4: Figure S4). The RF was the best model for mapping the probability of tick attachment, followed by GLM-net and GBT.

**Fig. 5:**
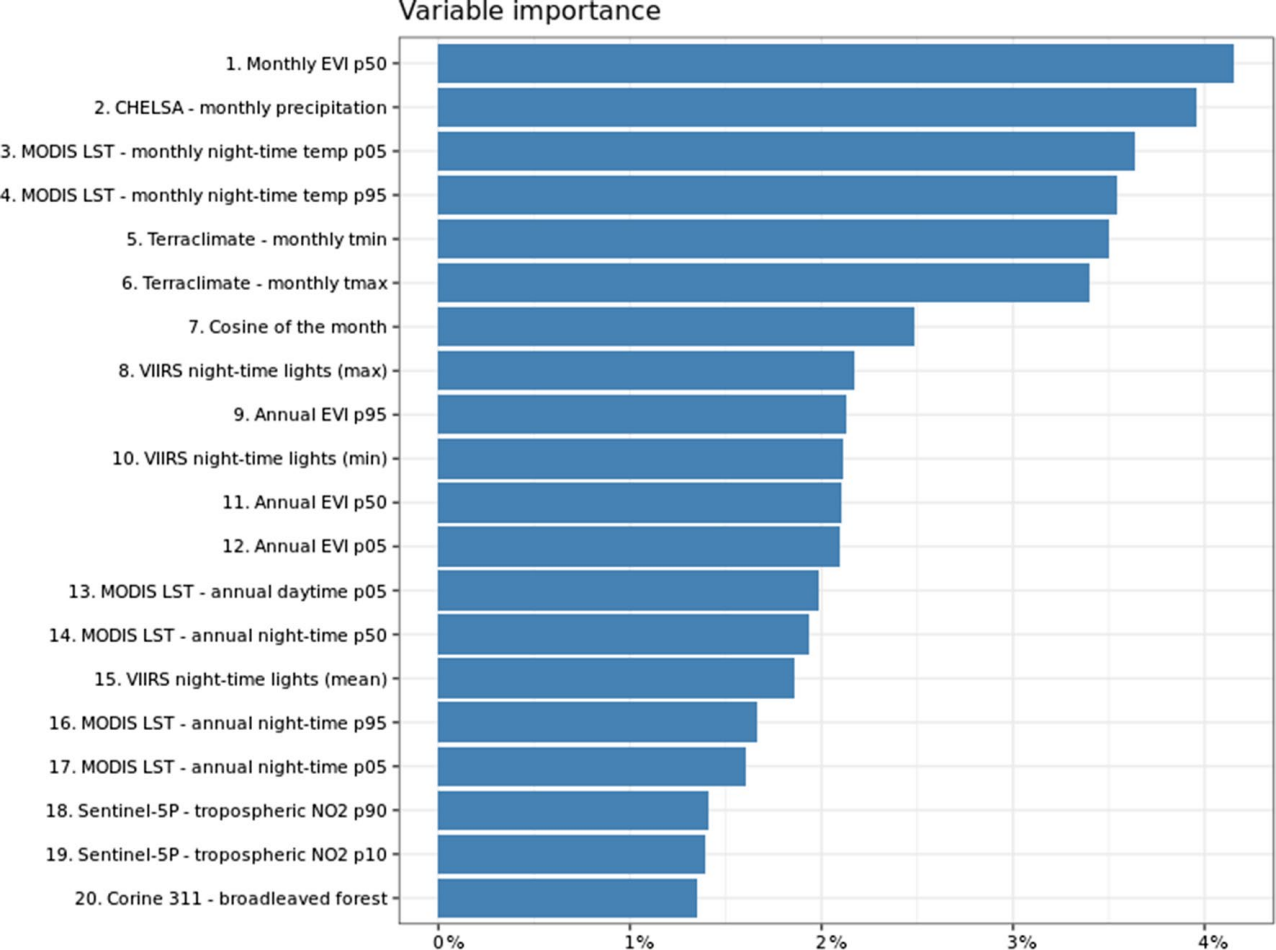
Variable importance plot of the top 20 covariates (out of 75) for the spatiotemporal risk for tick attachment in companion animals, based on the fitted Random Forest model. The sum of the variable importance for all covariates equals 1, but to ease the interpretation of results, we multiplied it by a hundred to be in percentages

**Fig. 6:**
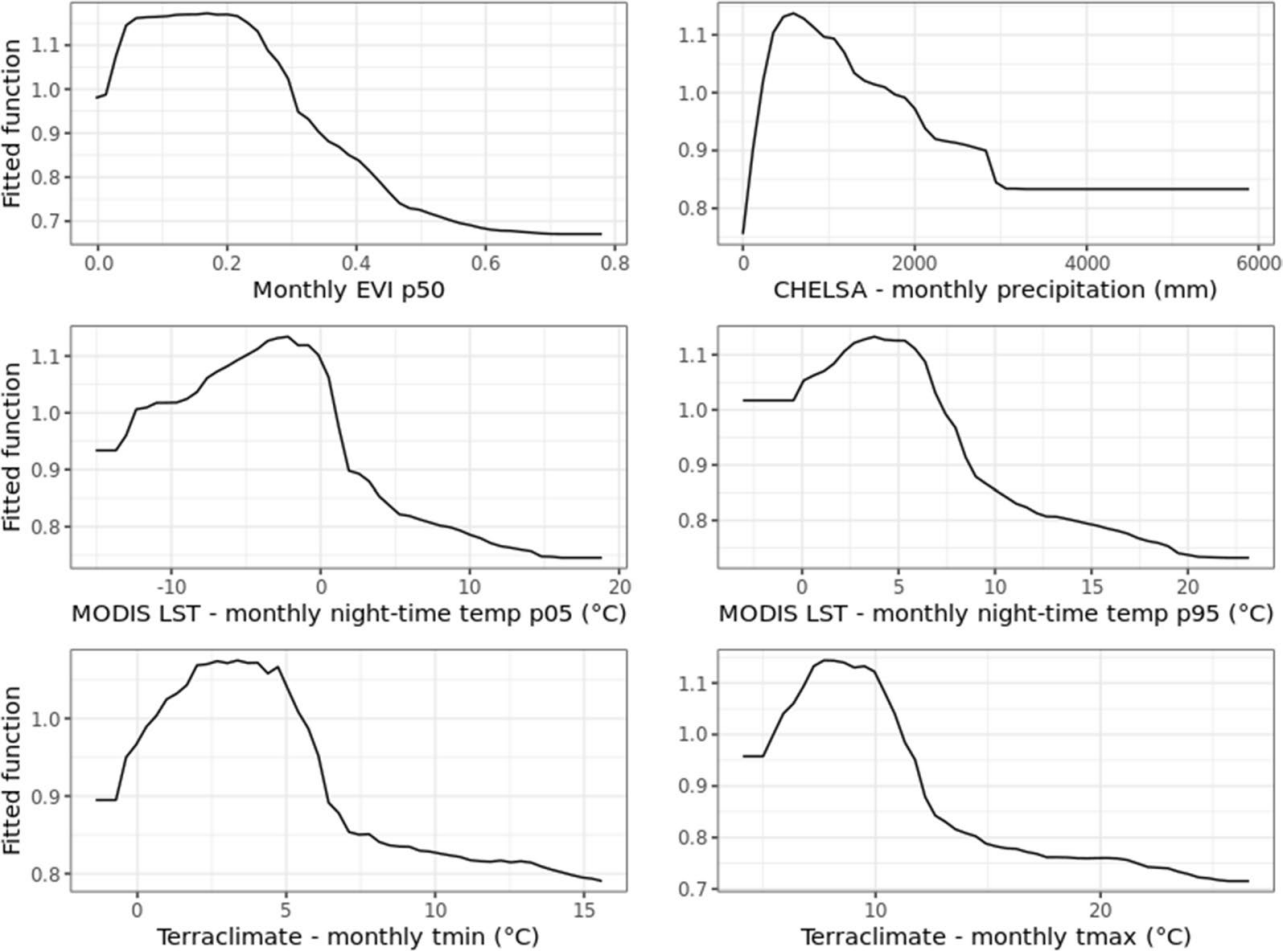
Partial dependence plots for the top six covariates (out of 75) for the spatiotemporal risk for tick attachment in companion animals, based on the fitted Random Forest model. EVI is the Enhanced Vegetation Index

The most relevant variables controlling the spatiotemporal risk for tick attachment for companion animals were the monthly mean EVI, followed by monthly precipitation, minimal and maximal monthly temperature, including nighttime temperature (Figs. 5 and 6). More precisely, PDP showed that green, dense vegetation, particularly woodland (coniferous and broadleaf), best explained the areas at risk for tick attachment. The probability of tick attachment grew sharply with an increasing EVI > 0.1 but decreased with a high EVI > 0.6. Notably, the high EVI shown in Fig. 6 is a rare feature, possibly due to missing training points, as veterinary clinics and pet owner residences are not typically inside nature parks or forests/woodland areas. In addition, risk areas for tick attachment were explained by monthly precipitation between 600 mm and 1200 mm, minimum monthly temperature between $-2\hbox {C}^{\circ }$ and $5\hbox {C}^{\circ }$, and maximum monthly temperature between $5\hbox {C}^{\circ }$ and $10\hbox {C}^{\circ }$, respectively. Similar results were obtained for the minimum and maximum monthly nighttime temperature, ranging between $-5\hbox {C}^{\circ }$ and $2\hbox {C}^{\circ }$ and between $2\hbox {C}^{\circ }$ and $10\hbox {C}^{\circ }$, respectively (Fig. 6).

**Fig. 7:**
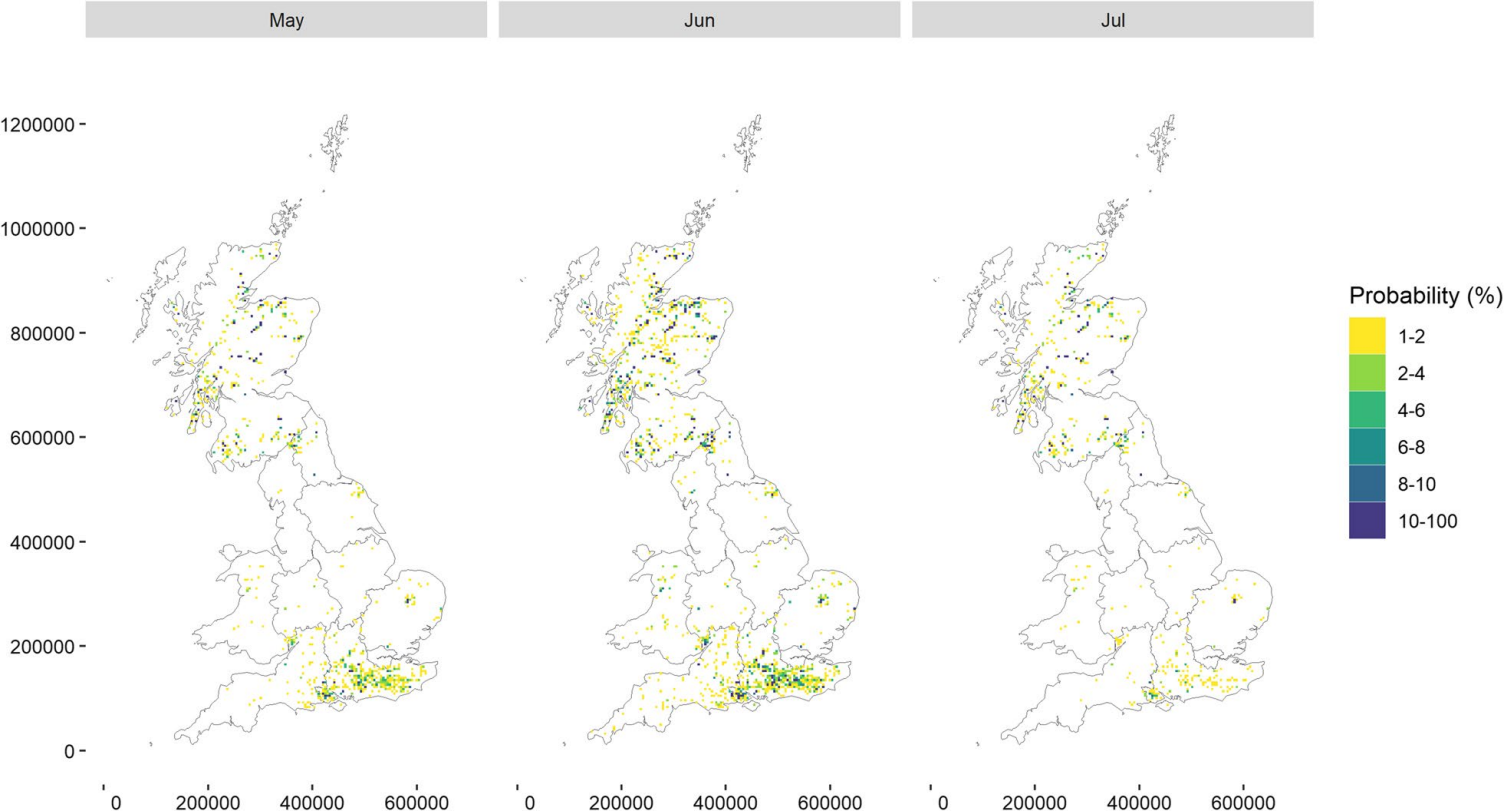
Long-term predicted spatial risk, probability (%) for tick attachment in companion animals, focusing on the peak period from May to July between 2014 and 2021 in Great Britain using SAVSNET data

The long-term monthly predictions for 2014–2021 showed marked seasonality of the spatial risk for tick attachment, with a peak period between May and July (Fig 7 and Rshiny interface) and no tick activity predicted from September to March. Exploring spatial trends showed that tick attachment risk corresponded mostly to grassland areas and forests (see Rshiny interface). More precisely, in June, the peak month of tick attachment, from a total of 21,391 predicted grid cells with a risk of tick attachment, 57% corresponded to broadleaf and coniferous woodland, followed by 25% of improved grassland. In northern parts of the country, suitable areas for tick attachment were predicted in mainly grid cells dominated by woodland: 89% (out of 659 predicted) in North East and North West England, 85% (out of 694 predicted) in Wales, 80% (out of 285 predicted) in Yorkshire and the Humber, 77% (out of 9528 predicted) in Scotland and 73% (out of 548 predicted) in the West and East Midlands. In the South East and West England and Greater London, out of 8810 predicted grid cells at risk for tick attachment, 52% were in improved grassland, followed by woodland (29%) dominated land. In East England, out of 867 predicted grid cells at risk of tick attachment, 54% were in woodland and 28% in horticulture/arable-dominated land.

Areas for tick attachment in June were predicted in the green and woodland areas in the Greater London region (e.g. Epping Forest, Richmond Park) and areas in South East England, in particular in areas of South, Central and North Hampshire (e.g. New Forest National park), East and West Surrey, East and West Sussex, Berkshire and central parts of Kent (e.g. Kent Downs); green and forest areas around Lydney in Gloucestershire (e.g. Mallards Pike) and around Salisbury in Wiltshire in South West England (e.g. Hazel Hill Wood Nature Reserve); forest patches around Thetford in the East Anglia in the East of England (e.g. Thetford Forest), forest areas around Scarborough in North Yorkshire (e.g. North Riding Forest Park), North East and West England such as larger forest areas on the borders between Northumberland, north Cumbria and South of Scotland (e.g. Kielder Forest Park) as as well as many scattered forest areas across Scotland, from the South up to the very North (Fig. 7 and Rshiny interface).

## Discussion

Understanding individual and spatiotemporal risk factors for tick attachment are a prerequisite if more targeted risk reduction of tick exposure and infestation is to be achieved. This is especially relevant in light of growing concerns about environmental contamination with parasiticides frequently used on pets. This study used a novel data source available through text mining of > 7 million EHRs from a large sentinel network of veterinary clinics across GB to identify individual host and environmental, climate, human-induced and host distribution factors for tick attachment in companion animals that can also be used as a proxy of risk to humans.

### Individual host risk factors for tick attachment

We found that tick attachment was more likely to be observed in animals 2 to 6 years of age, with those < 1 and > 10 the least likely to be observed with ticks [2, 3]. Variations in animal behaviour at different ages may confer variable risks of exposure to ticks and, thus, tick attachment. For example, younger dogs are more likely to be active explorers; likewise, younger cats are more likely to be hunters, while older animals possibly venture outside less frequently [70]. Younger animals are also recorded as visiting veterinary surgeons more regularly than their older counterparts [71], thus increasing the odds of noticing ticks during a veterinary consultation.

We also found that tick attachment was more likely reported in male and not neutered (entire) cats [3]. In the UK, most owned cats, such as those that make up the bulk of the SAVSNET database, likely manage their external access through the frequent use of cat flaps. As such, behavioural differences between the sexes may impact a cat’s exposure to the outside world. However, telemetry studies have shown little difference in the ranging behaviour of male and female cats, although they are relatively small studies in scale [24]. Our results are consistent with others that have suggested male cats are both more likely to be infested by ticks and at greater risk of carrying a TBD [3]. In contrast to cats, the sex and neuter statuses of a dog were not relevant predictors of tick attachment in our population, similar to findings of other studies in the UK [12, 72]. Unlike cats, most external access of dogs likely to attend the SAVSNET network is managed by their owners through organised walks, so tick exposure risk is perhaps more likely driven by the owner rather than inherent to the dog itself.

Regarding breeds, in dogs, a higher risk for tick attachment was associated with the pastoral breed group, possibly related to their hunting behaviour, that requires higher active outdoor exercise and thus higher odds for encountering ticks [2, 12]. In both cats and dogs, the risk was higher in crossbreed and unclassified breeds, possibly explained by differences in management and preventive care compared to owners of purebreds. We also observed that short-haired cats and dogs were less likely to present with recorded ticks than their long-haired counterparts, while some authors found that hair length had no significant association with tick attachment [3]. Pet owners may be more likely to notice and remove ticks on shorter-hair pets without visiting their vet and, therefore, less likely to contribute to the studied population. Short-haired animals may also be more effective at self-grooming and may remove ticks themselves, thus decreasing the odds for tick infestation [12].

### Spatiotemporal risk factors for tick attachment

In our study, tick records were noted throughout the year, with a peak in May in cats and June in dogs, corresponding to previous findings, both from SAVSNET [15], as well as from tick samples from cats and dogs identified with the TSS scheme [10, 13] and cross-sectional studies among small animal veterinary clinics [2, 3, 12]. This seasonal pattern was also predicted with the ensemble ML model.

The earlier peak of tick records in cats may reflect the activity of *I. hexagonus*, the most common tick species on cats, and the density and behaviour of its primary host, hedgehogs [8], which emerge from hibernation earlier in the year. In contrast, *I. ricinus*, the dominant tick species in dogs but also found on cats, spends most of its life off the host; thus, it is highly dependent on environmental conditions; it shows a more marked seasonality, with records increasing in April, peaking in May and June, and a marked reduction in August [7, 9].

Tick records from SAVSNET were noted throughout the country from the South of England, the Lizard Peninsula in Cornwall to North Eastern Scotland, and West Wales to East Anglia. Overall, tick records were observed across the country in spring and summer, while in autumn and winter, ticks were rarely reported in Scotland and Wales. Based on previous findings, we can only presume that our tick records reflect the spatial distribution of the two dominant tick species in companion animals [2, 10, 12, 13]. Both *I. ricinus* and *I. hexagonus* have a wide distribution throughout GB, with *I. hexagonus* more frequently observed in southern parts of the country [2, 3]. According to the latest TSS report, *I. hexagonus* records occur widely across England and parts of Wales, with notable areas around London and the south-east, Gloucestershire, the West Midlands and West Yorkshire. These often coincide with large urban conurbations where companion animals are likely exposed to urban *I. hexagonus* populations, and usually in areas where *I. ricinus* records are less common [10]. *Ixodes ricinus* are most abundant in woodlands, which provide adequate environmental conditions compared to other less suitable habitats [7, 9]. This may explain the difference between the predicted areas suitable for tick attachment in the southern and northern parts of GB obtained with our model; the woodland-dominated landscape—in the north—possibly reflecting *I. ricinus* attachment risk and the grassland-dominated vegetation type, followed by woodland in the south—possibly reflecting a mixture between *I. hexagonus* and *I. ricinus* attachment.

The EHR’s analysis further showed that the main drivers controlling the risk for tick attachment in companion animals are environmental and climatic. Highly suitable areas for tick attachment were predicted in grassland and woodland areas across the country, with moderate to high rainfall and monthly temperatures between $-5^{\circ }C$ and $10^{\circ }C$, environmental and climate conditions cited previously in the literature as important for tick survival, development and dynamics [59, 63] and also driving the human TBD risk in GB and Europe [65, 73, 74]. Ticks adapted to cold temperatures, such as *I. ricinus*, have been shown to commence questing at temperatures as low as $3^{\circ }\hbox {C}$ and the lowest temperatures that permit its metabolic function between $-5^{\circ }\hbox {C}$ and $-10^{\circ }\hbox {C}$ [75]. This may explain the predicted areas at risk across Scotland, which have colder weather than the rest of the country [58].

Overall, the ensemble ML model predicted suitable areas for tick attachment across several grassland and forest areas in GB, in particular areas in South East England and specific areas in South West England, East of England and Yorkshire as well as many scattered forest patches across Scotland (see Rshiny interface). Many of these areas have popular outdoor activity destinations, and some are known regional foci of Lyme disease, where the vector *I. ricinus* is also prevalent, such as New Forest, Salisbury Plain, Exmoor, South Downs, Thetford Forest and parts of Wiltshire and Berkshire in England and Wales, and the West Coast, Highlands and Islands of Scotland [76]. A visual comparison between our predictions and predicted areas of Lyme disease endemic foci in mainland Scotland suggests a possible overlap in several areas, such as Oban, Dumfries, Hamilton and Ayr [73]. Visually, similar overlaps were observed for Thetford Forest, New Forest National Park and woodland patches between Hampshire and Dorset in southern England, where the TBE virus was detected in ticks, followed by several human cases [77, 78]. Knowing that pet owners, whether of cats or dogs, are at increased risk of encountering ticks compared to households without a companion animal [6] and that TBE virus and *B. burgdorferi* can be transmitted rapidly following a tick bite [77, 79], SAVSNET, through its network of participating veterinary clinics, can further have a preventive role among pet owners to be tick-aware and take protective measures against tick bites when spending time outdoors, particularly in areas predicted at a higher risk for tick attachment and overlapping with foci of TBD [6].

 In the current work, cat and dog densities and deer probability of presence were not relevant drivers of the spatial risk for tick attachment. There are a few possible explanations for these results. The spatiotemporal model was trained based on records at a 1-km spatial resolution, taking as a possible place of exposure the vicinity of the pet owner’s residence [22]; the latter were mainly in suburban areas with a lower density of cats and dogs and a lower probability of deer presence in these grid cells and consequently affecting the results of our model.

Second, our results may also suggest the role of other hosts in the spatiotemporal risk of tick attachment. As mentioned before, *I. ricinus* has no pronounced host specificity and can feed on a range of host species, while *I. hexagonus* predominantly feeds on hedgehogs and other small mammals found throughout GB [8, 9].

Our results may also suggest that the spatial distribution of the risk for tick attachment in cats and dogs is controlled by a complex set of factors that include local conditions related to the habitat and interconnections between vegetation and climate that may affect tick survival and how the host population redistributes ticks with their movement rather than their number and presence/absence in a given grid area [80–82]. Though works suggest that companion animals may play a role in establishing and maintaining ticks in the peridomestic zone (garden) [82, 83], it can also be reasoned that companion animals could reduce tick survival in an enclosed habitat where pets are the prime food source for ticks if they are preventively treated against ticks [82]. Regarding the wild deer, we chose to work with one indicator to get a global overview of their role in the risk for tick attachment; however, future works should focus on better understanding the role of each deer species on the spatial risk of tick attachment, given their different distribution across GB and known role in maintaining and dispersion of tick populations in nature [84].

The SAVSNET network comprises 452 veterinary clinics, participating voluntarily, mainly from suburban areas and representing approximately 18% of the small animal veterinary clinics across GB. Most SAVSNET veterinary clinics are from South East and North West England. Therefore, our work has a selection bias because practices are not randomly recruited, making our findings difficult to extrapolate to all veterinary visiting cats and dogs in GB. Spatial bias can be countered to some extent by broadening the base of SAVSNET veterinary clinics in under-represented areas and considering the distribution of small animal veterinary clinics and companion animals in GB.

Furthermore, the SAVSNET clinical notes did not provide information on the location of the exact exposure to ticks of the animal. We thus used, for the modelling, the 1-km grid of the pet owner’s residence as an approximation of the most probable place of exposure, assuming that most tick attachments happen in the vicinity of the pet owner’s homes [22]. Clearly, however, some ticks will be picked up further from this location. Future work could consider varying buffer and grid sizes (2 km, 5 km) around the pet owner’s residence to assess whether the model performance increases.

Currently, SAVSNET EHRs hold very little information on the tick species associated with each tick record, only five out of 2000 tick records in a previous analysis by Tulloch et al. [15]. As a result, we cannot provide further accurate information, for example, on distribution changes for specific tick species in cats and dogs as provided with other schemes and studies [2, 3, 10]. A possible way to overcome this constraint is to encourage veterinarians to document information on tick species in the EHRs as much as possible.

Purposeful (active) sampling in veterinary practices in GB found that during the peak season of tick activity, over one in five examined animals was carrying a tick (30.7% of dogs and 32.4% of cats) [2, 3]. In contrast, our work relies on the passive recording of ticks by participating practitioners. In day-to-day veterinary practice, ticks on animals may go unnoticed or unrecorded, particularly if there is a more urgent clinical need during the visit. In addition, many companion animals will not visit a veterinarian because the owner is unconcerned, removed the tick themselves or was unaware of the ticks [15]. Therefore, the number of SAVSNET tick records used in our work in cats and dogs likely represents an underestimate of the true burden of infestation. Consequently, the resulting spatiotemporal predictions of the ensemble ML model should be interpreted carefully. The current predictions most likely underestimate the true tick attachment risk, and areas predicted as no or lower risk do not necessarily mean that the risk does not exist.

Future works on the SAVSNET data should explore other modelling options, for example, using only the tick (presence) records as a possible compromise between data quality and sensitivity of predictions. In this context, it should be envisaged to assemble complementary tick records obtained from various surveys and studies in GB, such as the TSS scheme and the Big Tick Project [2, 3, 10], to allow for a deeper understanding of the environmental, climate, human-induced and host distribution factors of tick attachment in cats and dogs and as a proxy of risk to humans.

## Conclusions

Our study provides valuable insight into the individual and spatiotemporal factors influencing tick attachment of cats and dogs and as a proxy for human risk in GB using Big data analytics of > 7 million EHRs. The spatiotemporal risk of tick attachment is shaped by the interplay of climate and vegetation type that exert a crucial role in the life cycle of ticks. These results are relevant from a veterinary and public health perspective, as pets and humans frequenting grassland and forest areas predicted with a higher probability for tick attachment should be made aware of the risk, especially in high-risk times of year. Recommendations can be further aligned with the known presence of TBD in the veterinary practice area, as well as each animal’s demographics and lifestyle, including regular ectoparasiticide treatment and tick checks. Tick records originating from passive surveillance systems such as SAVSNET should be incorporated into a workflow with other tick surveillance schemes in GB for a more complete understanding of the drivers of tick attachment that may elude monitoring and surveillance networks when data are analysed separately. Through its large network of participating veterinary clinics, SAVSNET can further have a preventive role among pet owners, reminding them to be tick-aware when spending time outdoors, particularly in areas predicted at a higher risk for tick attachment (see Rshiny interface). Finally, we provide all the covariates and the code for the spatiotemporal modelling to make this study fully reproducible.

### Supplementary Information

**Additional file 1: Figure S1.** Distribution of the SAVSNET participating veterinary clinics in Great Britain between 2014 and 2021.

**Additional file 2: Figure S2.** Kernel ratio of cat and dog records vs. all records from the SAVSNET network in Great Britain between 2014 and 2021.

**Additional file 3: Figure S3.** Relative risk of the cat and dog tick records (presence) vs. no tick records (absence) from the SAVSNET network in Great Britain between 2014 and 2021.

**Additional file 4: Figure S4.** Area under the ROC curve (AUC) of the Ensemble machine learning spatiotemporal model.

**Additional file 5: Table S1.** List of covariates used in the Ensemble machine learning spatiotemporal model.

## Data Availability

All input covariate layers, produced predictions and code used to fit and validate the spatiotemporal models are available at https://doi.org/10.5281/zenodo.7625174. The Rshiny interface of produced predictions and trends is available at https://opengeohub-msahin.shinyapps.io/GB_tickprobability.
