# Supplementary figures and images for: Risk factors for tick attachment in companion animals in Great Britain: a spatiotemporal analysis covering 2014–2021
Source: Parasit Vectors. 2024 Jan 22;17:29. doi: 10.1186/s13071-023-06094-4 (PMC10804489; doi:10.1186/s13071-023-06094-4)

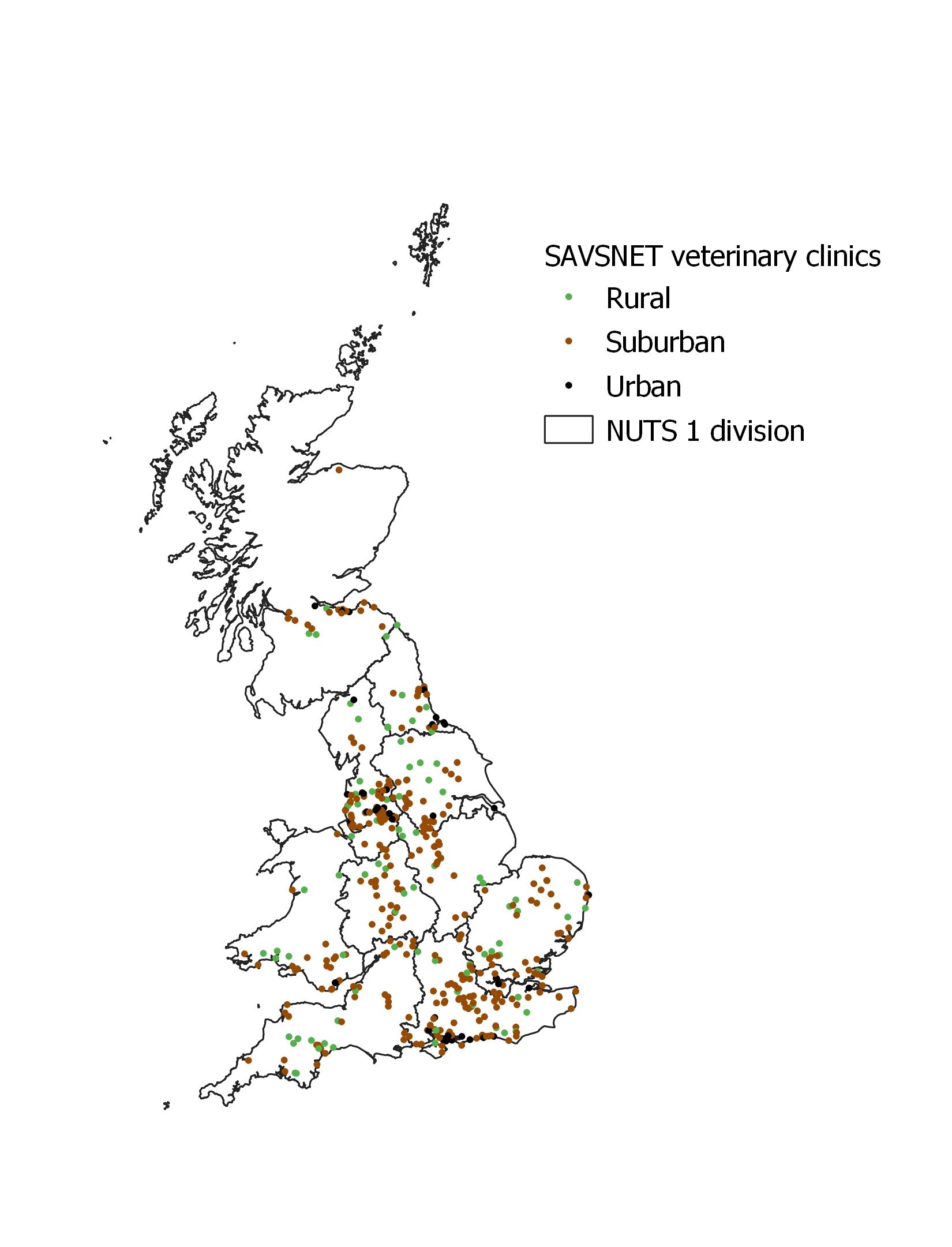

Supplement: Supplementary file 1 — Additional file 1: Figure S1. Distribution of the SAVSNET participating veterinary clinics in Great Britain between 2014 and 2021. [file 13071_2023_6094_MOESM1_ESM.png]

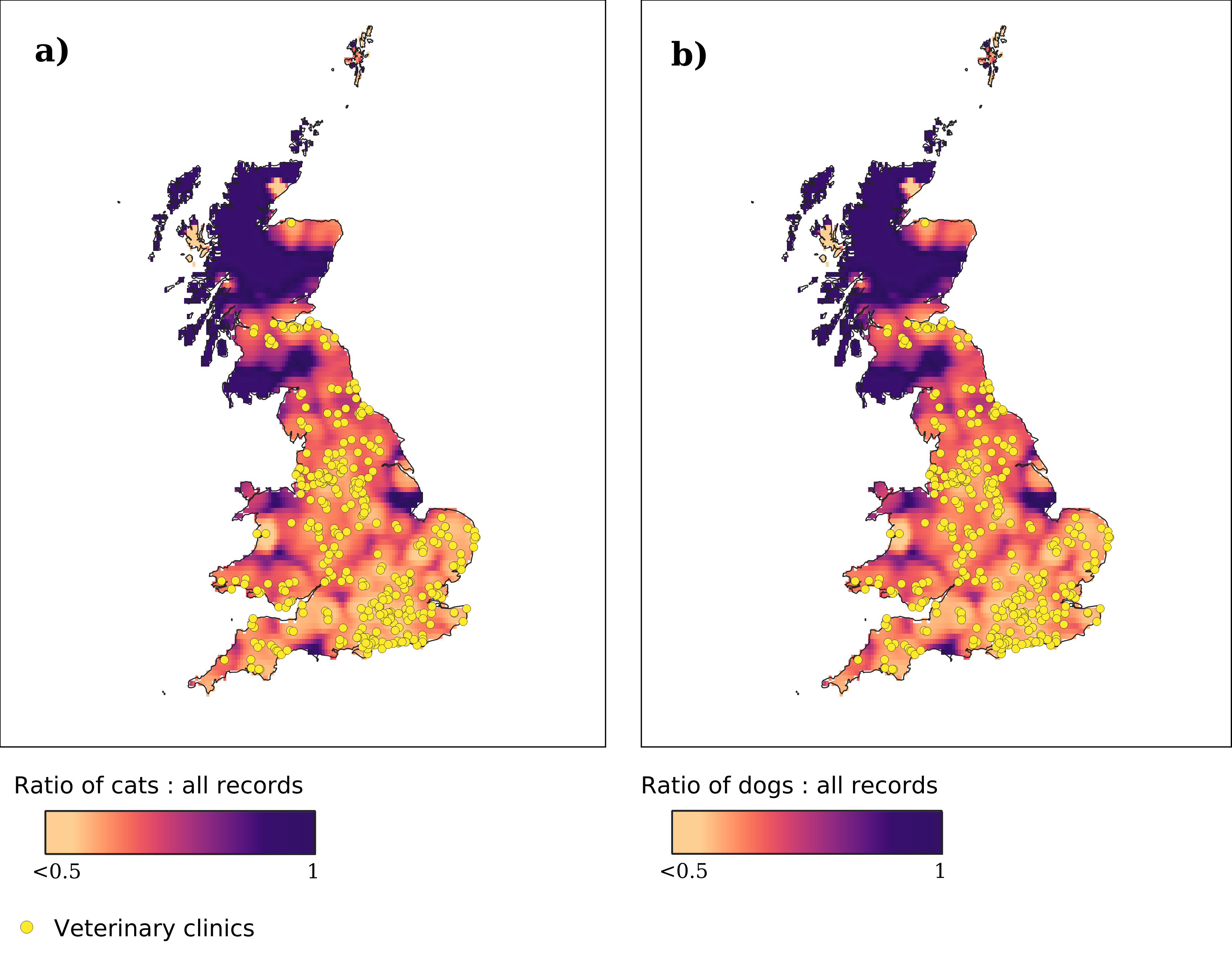

Supplement: Supplementary file 2 — Additional file 2: Figure S2. Kernel ratio of cat and dog records vs. all records from the SAVSNET network in Great Britain between 2014 and 2021. [file 13071_2023_6094_MOESM2_ESM.png]

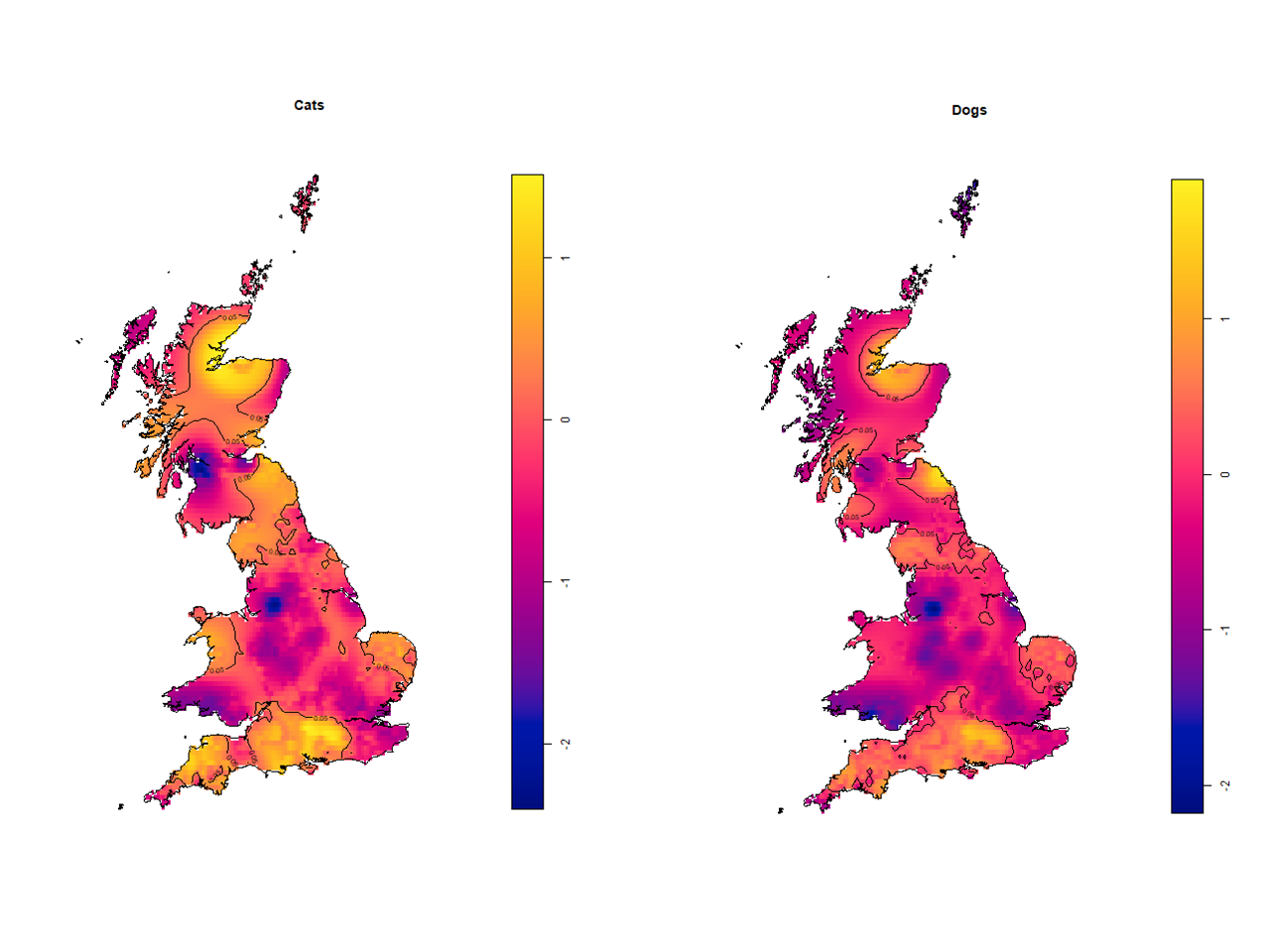

Supplement: Supplementary file 3 — Additional file 3: Figure S3. Relative risk of the cat and dog tick records (presence) vs. no tick records (absence) from the SAVSNET network in Great Britain between 2014 and 2021. [file 13071_2023_6094_MOESM3_ESM.png]

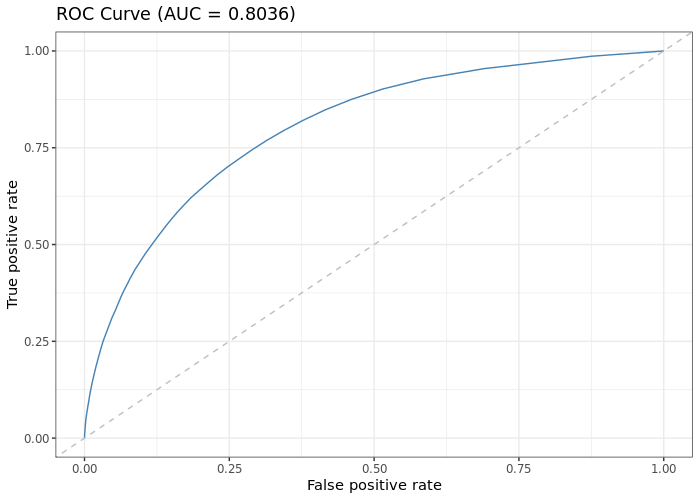

Supplement: Supplementary file 4 — Additional file 4: Figure S4. Area under the ROC curve (AUC) of the Ensemble machine learning spatiotemporal model. [file 13071_2023_6094_MOESM4_ESM.png]
